# Remote Temperature-Responsive Parafilm Dermal Patch for On-Demand Topical Drug Delivery

**DOI:** 10.3390/mi12080975

**Published:** 2021-08-18

**Authors:** Shahrukh Zaman Akash, Farjana Yesmin Lucky, Murad Hossain, Asim Kumar Bepari, G. M. Sayedur Rahman, Hasan Mahmud Reza, Shazid Md. Sharker

**Affiliations:** Department of Pharmaceutical Sciences, North South University, Dhaka 1229, Bangladesh; shahrukhzamanakash@gmail.com (S.Z.A.); farjanayesmin981@gmail.com (F.Y.L.); murad.hossain@northsouth.edu (M.H.); asim.bepari@northsouth.edu (A.K.B.); sayedur.rahman@northsouth.edu (G.M.S.R.); Hasan.reza@northsouth.edu (H.M.R.)

**Keywords:** remote control, temperature-responsive, dermal drug delivery, on-demand therapeutic effects

## Abstract

The development of externally controlled drug delivery systems that can rapidly trigger drug release is widely expected to change the landscape of future drug carriers. In this study, a drug delivery system was developed for on-demand therapeutic effects. The thermoresponsive paraffin film can be loaded on the basis of therapeutic need, including local anesthetic (lidocaine) or topical antibiotic (neomycin), controlled remotely by a portable mini-heater. The application of mild temperature (45 °C) to the drug-loaded paraffin film allowed a rapid stimulus response within a short time (5 min). This system exploits regular drug release and the rapid generation of mild heat to trigger a burst release of 80% within 6 h of any locally administered drug. The in vitro drug release studies and in vivo therapeutic activity were observed for local anesthesia and wound healing using a neomycin-loaded film. The studies demonstrated on-demand drug release with minimized inflammation and microbial infection. This temperature-responsive drug-loaded film can be triggered remotely to provide flexible control of dose magnitude and timing. Our preclinical studies on these remotely adjustable drug delivery systems can significantly improve patient compliance and medical practice.

## 1. Introduction

Drugs released from a drug delivery system (DDS) comply with the predetermined rate irrespective of patient need or changing physiologic condition [1]. In the physiologic system, an increase in temperature increases the blood flow on the skin, which can play a significant role in transdermal and dermal drug delivery. Furthermore, the average body temperature is about 37 °C (98.6 °F), but various conditions, illnesses, and medicines can raise body temperature. As the blood flow and drug distribution are related, a mild rising temperature (37 °C to 45 °C) can be used to increase drug circulation and therapeutic outcomes. Additionally, heat can be generated remotely on the skin through various typical tools including hot air, a heater, and light. Understanding these temperature effects and designing meaningful thermoresponsive materials are significant steps toward developing and evaluating a heat-responsive drug-delivery system to the skin. Recent studies on smart stimulus-active topical drug delivery systems, including microneedles and dermal patches, have demonstrated their potential merit over general topical products in terms of therapeutic efficacy and parent compliance [2,3].

Innovative materials have contributed to drug delivery systems (DDS) that can work at the site of disease [4,5]. Using a combination of therapeutic agents, stimulus-responsive materials impact every branch of medicine [6,7]. Several stimulus-responsive materials have been developed using temperature [8,9,10]. However, it is challenging to create thermoresponsive delivery systems that will be activated at the site of disease. Therefore, it is an excellent opportunity to combine heat-active materials with active pharmaceutical ingredients (APIs) to design remote temperature-controlled local drug release systems [11].

Thermoresponsive parafilm paper (PP) is one of the common materials typically used to seal containers. PP is composed of wax and polyolefin. Moreover, it is a malleable, nontoxic, tasteless, and odorless self-sealing thermoplastic material [12]. Biocompatible stimulus-responsive materials on drug delivery systems have already been established that dissociate or associate the drug on the basis of changes in temperature and pH, allowing drug release from the delivery system [10]. A remote thermally positioned heat-responsive PP can be employed in this scope to develop a stimulus-responsive local drug delivery system. The release of drugs can be regulated according to therapeutic needs and changing physiologic conditions [13].

Remotely thermal-controlled local DDS in conjunction with temperature-responsive material may bring new opportunities and directions in heat-responsive drug carrier development. The use of temperature-sensitive materials such as parafilm paper (PP) in dermal drug carriers remains mostly unexplored. By creating and characterizing drug-material conjugates, this study can allow controlling stimuli and developing applications for drug delivery at the site of disease. The integration of wax-based parafilm paper with these proposed systems is uniquely positioned to probe the application of an on-demand remote temperature-responsive delivery system.

## 2. Materials and Methods

### 2.1. Materials

Parafilm (PP) M rolls were obtained from Heathrow Scientific, Vernon Hills, IL, USA. Neomycin and lidocaine HCl were obtained from Incepta Pharmaceuticals Ltd., Dhaka, Bangladesh. A standard Band-Aid film from Johnson & Johnson (New Brunswick, NJ, USA) Band-Aid and Cardio green (Indocyanine Green, ICG) were purchased from Sigma Aldrich (Darmstadt, Germany).

### 2.2. Characterizations

A Xintest HT-02 Thermal Imaging Camera (Shenzhen, China) and a portable mini-heater (JC Trumps (50 W), China) were used. UV–Vis spectral absorptions were recorded using a UV-1800 Shimadzu Spectrophotometer (Kyoto, Japan). The TGA (thermal gravimetric analysis) and DSC (differential scanning calorimetry) measurements were performed according to an STA 449 F3 Jupiter test machine. The sample films were heated from 20 to 800 °C with 5 °C·min^−1^ in a streaming nitrogen atmosphere (purge: 50 mL/min; protective 20 min).

### 2.3. FT-IR Measurements

FT-IR (Fourier-transform infrared) measurements using ATR (attenuated total reflection) of the samples were carried out in transmission mode in the region from 400 to 4000 cm^−1^ using an infrared (IR) Shimadzu spectroscope, iR Affinity 1 (Shimadzu, Kyoto, Japan). The spectra were measured at 2 cm^−1^ resolution. The bare PP film, dye loaded PP film, and drug-loaded PP film samples were placed directly in the spectrometer.

### 2.4. Scanning Electron Microscopy (SEM) Imaging and EDX (Energy-Dispersive X-ray) Spectroscopy

Morphology and surface features of suture thread were studied using FE-SEM (field-emission scanning electron microscopy) instrument Model JSM-6700, JEOL Ltd., Tokyo, Japan. Samples were prepared by cutting the film into 1 inch long sections on aluminum foil. The film samples were sputter-coated with gold–palladium alloy to boost electrical conductivity, thereby obtaining a high-resolution SEM image and EDX (energy-dispersive X-ray) spectrum for composition analysis. All SEM images were taken at either 5 kV or 15 kV.

### 2.5. Preparation of the Drug-Loaded PP

An active pharmaceutical ingredient (API) or a dye (model drug) was loaded on parafilm paper (PP) by dissolving the sample in a nonpolar organic solvent. In brief, the API (lidocaine or neomycin) or ICG (Indocyanine Green) dye solution was prepared with toluene, and the prepared sample (1 mg/mL) was added dropwise on parafilm paper PP (1 inch × 1 inch). The sample-loaded films were dried for 30–60 min. The unloaded dye or drugs and residue solvent were removed by washing PP film with deionized water. Finally, the functional parafilm paper was vacuum-dried completely for further experiments. The drug loading, as well as the PP thickness, surface, and material properties, was determined by UV spectroscopy, ATR-IR spectroscopy, scanning electron microscopy (SEM), TGA (thermogravimetric analysis), and DSC (differential scanning calorimetry) analysis.

### 2.6. Release Kinetics of Drug from Thermoresponsive PP

The loading content and loading efficiency were determined using a digital balance machine. The PP film in different treated conditions (loaded and unloaded) was accurately weighed, where the number of samples exceeded five. The following equations were used: loading content = ((drug-loaded PP film − bare PP film weight) × 100/(drug-loaded PP film weight); loading efficiency = (added drug (total) on PP film − deposited drug on PP film (after washing) × 100/added drug (total) on PP film).

The release behavior of API from drug-loaded PP was evaluated at 37 °C, 45 °C, and 55 °C in PBS (phosphate-buffered saline) (pH 4.0/5.5/6.5) in a shaking incubator. The chambers were immersed in 30 mL of release medium (PBS) at 37 °C, 45 °C, and 55 °C. Finally, the samples (1 inch × 1 inch) were placed in a shaking incubator for the whole experimental period (24 h). To determine the amount of drug released, a 3 mL solution was taken from the outside released medium, and absorbance was measured at the selected λ_max_ (lidocaine = 263 nm; neomycin = 262 nm) using UV–visible light spectroscopy and a standard curve; the amount of drug released was then calculated [14,15].

### 2.7. In Vivo Studies

The animals were housed under standard laboratory conditions (relative humidity 55–65%, room temperature 23 ± 2 °C, 12 h light/dark cycle). Adult Swiss albino mice weighing 35–45 g of either sex were obtained from the Department of Pharmaceutical Sciences animal house, North South University. The animals were fed with a standard diet and water ad libitum. The University Animal Research Ethical Committee approved the experimental protocol. Under ketamine anesthesia, 2 mm long, 4–5 µm depth full-thickness incisions were made on the left dorsal flank, followed by wound closure with either bare PP or drug-loaded PP using standard patch techniques [16]. The mice were also remotely heated gently for 5 min (at 45 °C) at the patch site using a mini-heater after patch implantation. The healing process/therapeutic effects around the wound was visually inspected and documented over 7 days using both visible observations and photographic images. A digital slide caliper quantified the average incision length near the stitch point.

The drug (for example, anesthetic lidocaine) inducing anesthesia was evaluated in the animal closing skin wounds by subjecting them to a pinch test. The pinch tests performed in this work were adopted from an established method.

A single, blinded investigator conducted every test to minimize variation in examination and bias in observation [14,16]. The test (for example, analgesia) procedure was performed using curved forceps to apply a pinch in different locations within the 1 cm^2^ area of complete wound sealing. The animal response for every pinch was recorded. The optimum care was taken to ensure constant force, and this was maintained as low as possible. The effectiveness of a topical antibiotic (neomycin) was evaluated in terms of wound healing and the closing surgical incision rate. All animals were tested 0.5, 1, 2, and 3 h post surgery and then every day for the next 7 days.

### 2.8. Histological Analysis

The histological (H&E staining) analysis was applied to the wound surface’s skin tissue area recovered after the seventh day of treatment. The staining protocols were adopted from our previous report [17]. The mice were sacrificed for tissue collection using a standard anesthetics procedure, and the tissue was collected from the stitch site. Finally, the tissues were examined at 10× magnification with a BB.1153-PLi optical microscope (Euromex Microscopen B.V. Arnhem, The Netherlands).

## 3. Results and Discussion

The findings from this research project can be used to generate a remote-controlled thermoresponsive patch focused on dermal drug delivery and local therapeutic effects. The investigational results obtained from this parafilm paper (PP) study can create new technological tools under variable temperature to maintain on-demand drug release and minimize discomfort from inflammation, bleeding, microbial infection, and other skin diseases.

**Scheme 1:**
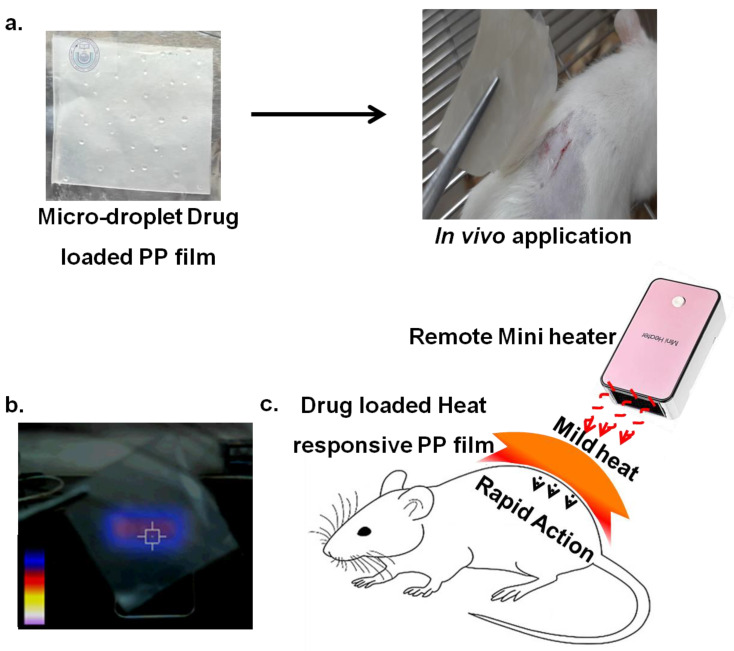
(**a**) Image of microdroplet drug (lidocaine) solution on PP film (1 inch × 1 inch), where complete drug loading can be achieved following a simple drying process, thus representing the proposed remote temperature-responsive local DDS for in vivo studies on dermal areas. (**b**) External portable mini-heater (50 W) applied to the thermo-responsive drug-loaded PP film for mild heat measurements. (**c**) Schematic representation showing the heat-responsive in vivo application of a drug-loaded PP film patch.

Toward this goal, the therapeutic drugs were loaded on PP film by coating a drug (lidocaine or neomycin) solution. The complete experimental design and proposed application method are shown in Scheme 1a. Like microneedle drug delivery, we expect that the ordered array (6 × 6 drops per square inch) of the microdroplet patch could enhance the penetration of drugs directly into the skin layers. As the PP carrier is temperature-responsive, its malleable properties can be applied to control drug release from the film [18]. The required mild heat can come from body temperature and/or an external portable mini-heater. Our proposed scheme for this promising application is shown in Scheme 1b,c.

**Figure 1:**
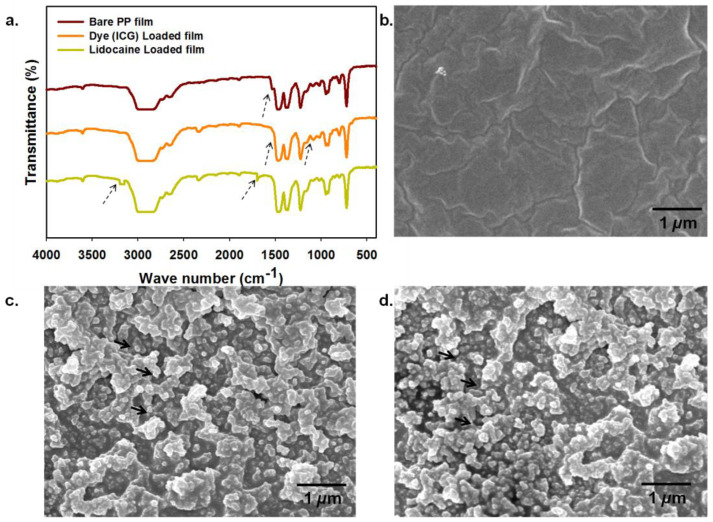
(**a**) The FT-IR transmission peaks of bare PP film, dye (ICG)-loaded PP film, and lidocaine-loaded PP film. The scanning electron microscopic (SEM) images (scale bar 1 μm) of (**b**) bare PP film, (**c**) dye (ICG)-loaded PP film, and (**d**) lidocaine-loaded PP film, where the arrow shows the site of loading in the PP film.

The surface-coated ICG dye (as a model drug) and lidocaine on PP film were evaluated using FT-IR (ATR mode) spectroscopy analysis (Figure 1a). The bands at 717 and 728 cm^−1^ were contributed by parafilm C–H rocking, whereas those at 1367 and 1378 cm^−1^ represented parafilm C–H bending, and those at 2847, 2874, 2916, and 2950 cm^−1^ represented parafilm C–H stretching [19]. On the other hand, the characteristic peaks at 1400–1500 cm^−1^ and 900–1100 cm^−1^ of ICG were attributed to C=C stretching and vinyl stretching, respectively [7]. Additionally, the IR spectrum of lidocaine-loaded film showed bands around 1682 cm^−1^, representing C=O amide groups, whereas bands at 3180 cm^−1^ corresponded to N–H and C–H stretching in lidocaine [14]. Some of the peaks representing the chemical/functional groups of PP film coated by dye or drug disappeared, e.g., 1550 cm^−1^, with new peaks emerging that represented the coated dye or drug. ATR FT-IR allows measuring the chemical nature of the outermost coated surface. Therefore, many IR spectroscopic peaks were merged, thus occluding parafilm peaks.

The surface morphology of the bare PP film, dye (ICG)-loaded film, and lidocaine-loaded PP film was examined using a scanning electron microscope (SEM) (Figure 1b–d). Surface roughness was visible on both the dye-loaded and the drug-loaded PP film, whereas a smooth surface was observed on the bare PP film [20]. The light/dark contrast with noticeable microparticles denote clumps on the PP film surface that may have occurred due to the loading of the deposited dye or drug (lidocaine). The SEM images recorded for the bare PP film and drug-loaded PP film highlight the successful modification of the PP film surface (Supplementary Figure S1). The material composition of the sample-loaded PP films was further studied using EDX spectroscopy. The observed sample-loaded composite materials demonstrated 54.82% carbon and 19.44% oxygen on the surface, which was substantially higher than bare unloaded PP film (Supplementary Figure S2). There was a higher percentage of C and O atoms on the surface of the ICG-loaded PP composite film [21].

Moreover, the lidocaine loading content and loading efficiency on the PP film surface per square inch were found to be 3.50% and 95%, respectively. At the same time, the neomycin loading content and loading efficiency on the PP film surface per square inch were found to be 3.65% and 96%, respectively. Although depositing a liquid sample onto a PP surface helps to develop a drug-loaded film, it requires a subsequent drying process. The effect of drying the PP film necessitates further study. Additionally, the surface loading and the liquid nature of the organic medium may have been the reason for the change in PP film morphology.

**Figure 2:**
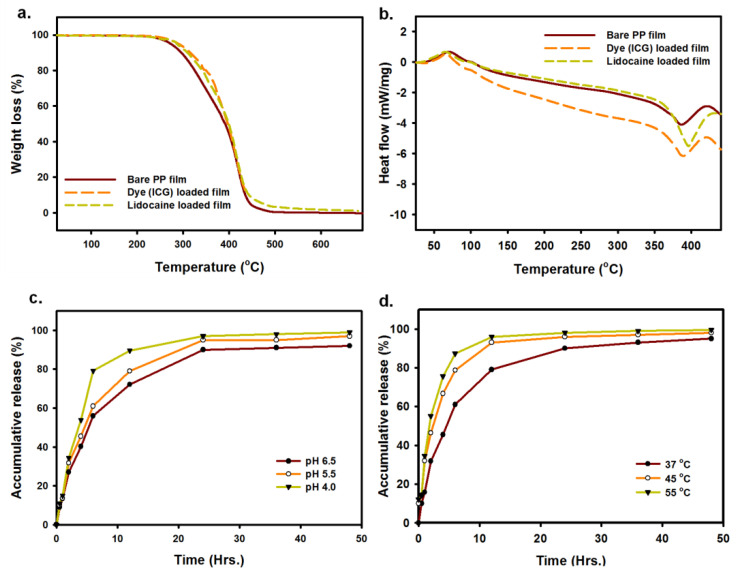
(**a**) TGA (thermogravimetric) studies of bare PP film, dye (ICG)-loaded film, and lidocaine-loaded PP film. (**b**) DSC analysis of bare PP film, dye (ICG)-loaded film, and lidocaine-loaded PP film. (**c**) In vitro pH-dependent (%) cumulative release of lidocaine from drug-loaded PP film at pH 4.0, 5.5, and 6.5. (**d**) In vitro temperature-dependent (%) cumulative release of lidocaine from drug-loaded PP film at 37 °C, 45 °C, and 55 °C at pH 5.5.

The thermal decomposition behavior of bare PP film, dye (ICG)- loaded film, and lidocaine-loaded PP film was evaluated using TGA (thermogravimetric) analysis. As shown in Figure 2a, the mass loss ratio of bare PP film started to decrease around 300 °C, roughly equivalent to that of dye-loaded PP film and lidocaine-loaded PP film [22]. This indirectly proves the homogeneity of the PP film with the loaded sample and the accuracy of the measurement techniques.

Figure 2b shows the typical DSC curves of bare PP film and dye (ICG)-loaded PP film, and lidocaine-loaded PP film. These DSC curves represent reference data that allow evaluating the changes in thermal properties depending on the nature of the PP film. It can be seen that there were two peaks in the DSC curve of the bare PP film. The sharp or prominent peak represents the solid–liquid phase change of the paraffin (melting temperature Tm ≈ 400 °C), and the minor peak to the left of the principal peak corresponds to the solid–solid phase transition of PP film (transition temperature Tt ≈ 50 °C) [23]. All studied PP films showed similar DSC curves, indicating that the process of coating dye or drug (lidocaine) onto the PP film was mainly a physical interaction with a minor chemical reaction. Since the drug/dye was deposited on the surface, the solid–solid phase change and solid–liquid phase change were only slightly altered in the dye- and drug-loaded parafilm.

The flexibility of externally controlled drug release from a drug carrier can improve the therapeutic outcome to meet pathologic needs. To investigate the release kinetics of drug (lidocaine) from PP film, we first studied the release behavior of lidocaine in PBS with different pH values corresponding to different dermal environments (pH = 4.0, 5.5, and 6.5), at 37 °C (Figure 2c) [24]. Secondly, we studied the release behavior at different temperatures, 37 °C, 45 °C, and 55 °C, at pH 5.5 [25]. The released lidocaine was collected at different time intervals to evaluate the pH-responsive and temperature-responsive drug release. Without remote heating, drug (lidocaine) release was moderately affected in response to pH changes. In the early stages, the drug was released slowly, and, after 6 h, 50–80% was released at pH 4.0 and 6.5. Nearly 65–85% of the lidocaine was released after 12 h in acidic media (pH 4.0 and 6.5) resembling the skin environment. However, the slightly variable drug release increases in more acidic media are them favoring solubility. To estimate how rapidly the drug (lidocaine) is released from PP film, we measured the release profile at different temperatures of 37 °C, 45 °C, and 55 °C, at pH 5.5 (normal skin environment). Since the PP film is inherently thermoresponsive, being soft and malleable at elevated temperature, the loaded drug (lidocaine) exhibited rapid release (~10% initially), with 60% being released 37 °C, 80% being released at 45 °C, and 90% being released at 55 °C, at pH 5.5, within 6 h of observation (Figure 2d). Elevated temperature induces the meltable property of PP film, thus releasing the coated drug from the surface [26]. Parafilm^TM^ is soft and sticky at about 54–66 °C, which can stimulate the release of drugs from the solid PP (parafilm) surface. The observed release was linked to an increase in diffusion of the drug following the combination of a mild increase in heat and pH [27].

Additionally, it was observed that the release after 24 h remained constant. This might have been due to the saturation of accumulated drug in the release medium. Similar release patterns were observed for a hydrogel-based dermal patch developed using alginate and NIPAM. The thermoresponsive behavior showed greater sensitivity to an increase in temperature [25]. Therefore, such a delivery system can be regulated externally. This functional externally controlled temperature-sensitive release system is expected to profoundly impact many therapeutic fields, including local anticancer drug delivery.

**Figure 3:**
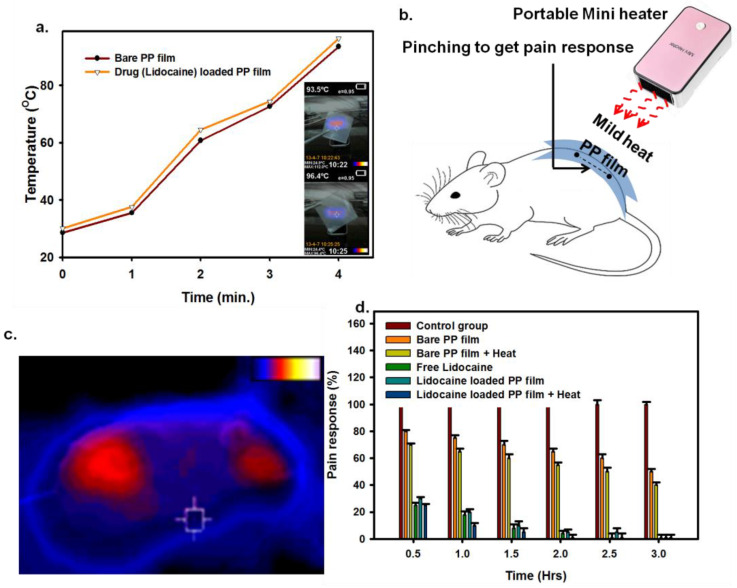
(**a**) Time-dependent heat generation curve of bare PP film and drug-loaded PP film as a function of external mini-heater. The inset images show the portable mini-heater (50 W)-induced thermographic image of bare PP film and drug-loaded PP film after 4 min of continuous observation. (**b**) Schematic image of pinching test model on mice. (**c**) Thermo-graphic image used to monitor temperature on mouse skin treated with drug-loaded PP film using a portable mini-heater to maintain a constant temperature of 45 °C. (**d**) Percentage (%) pain response in mice treated with bare PP film, bare PP film with heat, free lidocaine, lidocaine-loaded PP film, and lidocaine-loaded PP film with heat. Mild heat was induced by an external mini-heater (50 W) for 5 min to maintain a constant temperature of 45 °C. The pinching tests for pain response were evaluated 0.5 to 3.0 h after 5 min treatment at 45 °C.

As the PP film is generally soft and meltable under mild heat treatment, we selected the PP carrier for our thermoresponsive DDS. Following the application of mild heat, both bare PP film and drug-loaded PP film showed a similar rise in temperature, increasing by more than 95 °C within 4 min. The PP film temperature can be optimized using a portable mini-heater depending on the requirements. This observation also suggested that, at 95 °C or above, the PP film remained physically stable, making it suitable as a drug carrier (Figure 3a).

Local anesthesia has been used achieve sedation with many therapeutic benefits, including postoperative surgical treatment and large-volume parenteral delivery [14]. In this view, lidocaine and lidocaine-loaded PP films were used to evaluate the pain relief response. Pinch tests used the sedation depths of applied PP film at the skin incision site. The mice with the bare PP film and heat-treated PP film showed a 50–60% increase in pain relief response compared to the control group (Figure 3d). The considerable analgesic effects of the lidocaine-loaded PP film were observed within 30 min of treatment, and the maximum outcome was recorded after 1.50 h. In another group, where mild heat at 45 °C was maintained on the applied lidocaine-loaded PP film for 5 min, the highest sedation was observed 1 h post treatment (Figure 3c,d). The externally applied heat, thus, favored drug release, allowing pain tolerance to pinching. Moreover, the application of mild temperature can stimulate local blood flow, which can support a better therapeutic response to drug molecule release. However, the fundamental factors underlying this perspective need to be further studied. The results may have been a combined effect of the released lidocaine and the mild heat (45 °C), since the mild generation of temperature has a natural sedative effect on skin [28].

**Figure 4:**
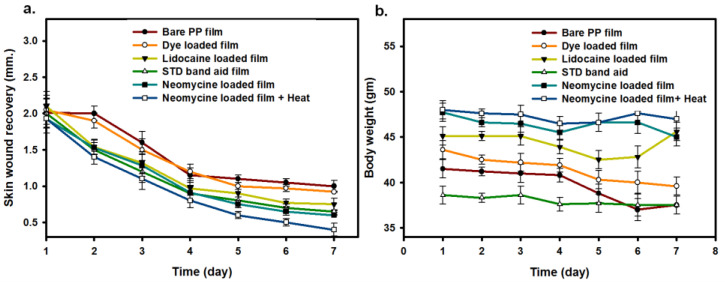
(**a**) Surgical incision average length (mm) and closure observed from day 1 to day 7 using bare PP film, dye (ICG)-loaded film, lidocaine-loaded film, neomycin-loaded film, STD (standard) Band-Aid film, and neomycin-loaded film with 5 min mini-heater treatment. The average incision length was quantified using digital slide calipers near the wound site. The mice were externally heated at 45 °C for 5 min in the heat groups (mean ± SD, *n* = 5). (**b**) The average body weight of treated mice when monitoring health status during in vivo studies.

As mentioned at the beginning of the investigation, the use of a remote temperature-controlled on-demand, dermal DDS can minimize topical discomfort from inflammation, bleeding, microbial infection, and so on [29]. Therefore, the PP was also loaded with topical antibiotic neomycin to evaluate the potential of local wound healing [30]. The healing process was observed for bare PP film, dye (ICG)-loaded PP film, lidocaine-loaded PP film, neomycin-loaded PP film, STD (standard) Band-Aid film, and neomycin-loaded PP film with 5 min 45 °C treatment on mouse skin from the day of surgical skin closure for 7 days (Figure 4a). In both drug-loaded PP films and the STD band-aid film, the healing process showed an almost similar trend, whereby a distinguishable recovery was observed. A similar result was noticed in neomycin-loaded nanofiber mats [30], where the neomycin-loaded mats led to better wound healing after tissue damage.

The complete recovery was also evaluated after 7 days of treatment from the external heat-treated neomycin-loaded PP film [31]. The average incision length quantified from digital images captured near the incision point shows the healing process and continuous decrease in size (mm) of the incision at 3 days, 6 days, and 7 days of observation (Supplementary Figure S3). All mice had the same incision depth (2 mm) and were administered a bare PP film or drug-loaded PP film with 5 min remote heating at 45 °C in these experiments. However, it is difficult to determine the local concentration of residual released drugs in tissue or blood, as it is not the desired goal of local applied medicine to be available in systemic blood circulation [32,33]. To investigate the physiological consequences of local treatments of PP film on mouse skin, we observed body weight changes following treatment (Figure 4b) [34]. The local application of bare or drug-loaded PP film and stimulation with mild heat did not cause any appreciable change in body weight in the treated group when comparing the first and days of observation. The mice tolerated the treatment well with no profound weight loss.

**Figure 5:**
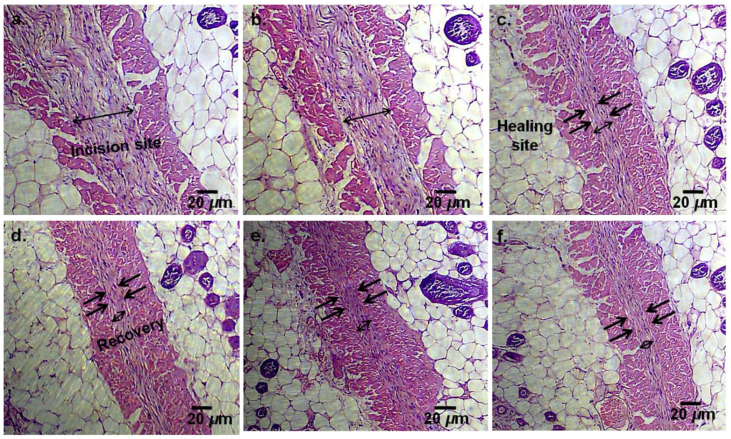
Histological (H&E staining) analysis obtained from the skin tissue area of the wound surface recovered after 7 days of treatment. Photomicrographs obtained from H&E staining of the skin wound area on day 7: (**a**) bare PP film, (**b**) dye (ICG)-loaded PP film, (**c**) lidocaine-loaded PP film, (**d**) neomycin-loaded PP film, (**e**) standard Band-Aid film, and (**f**) neomycin-loaded PP film with 5 min of portable (50 W) mini-heater (45 °C) treatment. The arrows indicate wound healing sites and wound closure areas. The scale bars represent 20 μm.

The tissue regeneration and wound healing processes were studied using H&E (hematoxylin and eosin) staining protocols adopted from our previous report [17]. The mice were sacrificed using a standard anesthetics procedure for tissue collection from the skin healing site. Figure 5 shows the histopathological profile of wounds treated with bare PP film, dye (ICG)-loaded PP film, lidocaine-loaded PP film, neomycin-loaded PP film, standard Band-Aid film, and neomycin-loaded PP film with 5 min of mild heating, on day 7 post treatment. On day 7, the lidocaine-loaded PP film, neomycin-loaded PP film, standard Band-Aid film, and neomycin-loaded PP film with mild heat group showed thicker and better healing, whereby the wound incision site was observed to be closed. In contrast, the bare PP film and dye-loaded PP film groups demonstrated inferior healing [35,36]. Moreover, compared with the no-heat group, the mild heat treatment group showed better and earlier recovery from the wound. Therefore, it can be concluded that mild temperature can stimulate the tissue to undergo the first stage of the repair process [28,37,38,39]. The results also demonstrated that the drug-loaded PP film significantly impacted the dermal healing process, and this effect was improved using remote mild heating.

## 4. Conclusions

New local drug delivery systems are being studied to improve patient compliance and address the shortcomings of systemic delivery. Accordingly, this study demonstrated a temperature-responsive PP film, which can control the release of drugs locally to boost the therapeutic effect. The simplicity of the microdroplet drug coating and the PP film properties can facilitate the continued development of local drug delivery systems. Our FT-IR, SEM, EDX, TGA, DSC, and thermograph results demonstrated the drug-loaded PP film surface, along with its thermal properties, as well as its ability to induce mild temperature-mediated wound healing. As a local anesthetic, the lidocaine-loaded PP film enabled area-specific analgesia. The antibiotic (neomycin)-loaded PP film also demonstrated wound healing potential, whereby the maximum recovery was observed as a function of mild hyperthermia. Therefore, virtually any drug can be loaded for a local therapeutic effect that can be stimulated by remote heating. In smart drug delivery, these user-friendly remotely controlled local therapeutic effects can significantly impact drug efficacy and medical practice.

## Data Availability

The data used to support the finding of this study are included within the article.

## Supplementary Materials

The following are available online at https://www.mdpi.com/article/10.3390/mi12080975/s1, Figure S1: The SEM images of PP film (control) treated with organic solvent (without drug) used for sample preparation (scale bar 1 μm). Figure S2: The EDX spectrum and composition ratio table of (a) bare unloaded PP film and (b) Dye (ICG) as a model drug loaded PP film. Figure S3: The images of full-thickness incision on a mice skin immediately after closure with Bare PP film, Standard Band-Aid film, Lidocaine loaded PP film, Neomycin loaded PP film, and Neomycin loaded PP film with two minutes mini-heater treatments, respectively. The digital image shows recovery after day 1 to day 7 of different treatments group (scale bar 1 cm).
