# Supplementary material for: Remote Temperature-Responsive Parafilm Dermal Patch for On-Demand Topical Drug Delivery
Source: Micromachines (Basel). 2021 Aug 18;12(8):975. doi: 10.3390/mi12080975 (PMC8400996; doi:10.3390/mi12080975)
Supplement: Supplementary file 1 [file micromachines-12-00975-s001.zip › micromachines-1307374-supplementary.pdf]

Electronic Supporting Information

Title

Remote temperature-responsive parafilm dermal-patch for on demand topical drug delivery

Authors

Shahrukh Zaman Akash [1], Farjana Yesmin Lucky [1], Murad Hossain[1], Asim Kumar Bepari[1], G. M. Sayedur Rahman[1], Hasan Mahmud Reza[1] and Shazid Md. Sharker [1,*]

Affiliations

[1] Department of Pharmaceutical Sciences, North South University, Dhaka-1229, Bangladesh.

*** Corresponding author**

Shazid Md. Sharker
Department of Pharmaceutical Sciences, North South University
Dhaka-1229, Bangladesh
Office: +880-2-55668200, Ext: 6402
E-mail:shazid.sharker@northsouth.edu

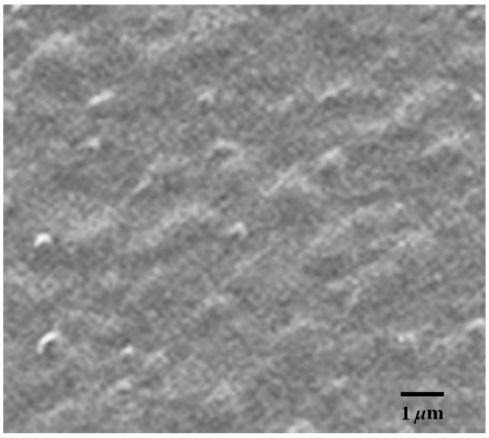

Figure S1. The SEM images of PP film (control) treated with organic solvent (without drug) used for sample preparation (scale bar 1 μm).

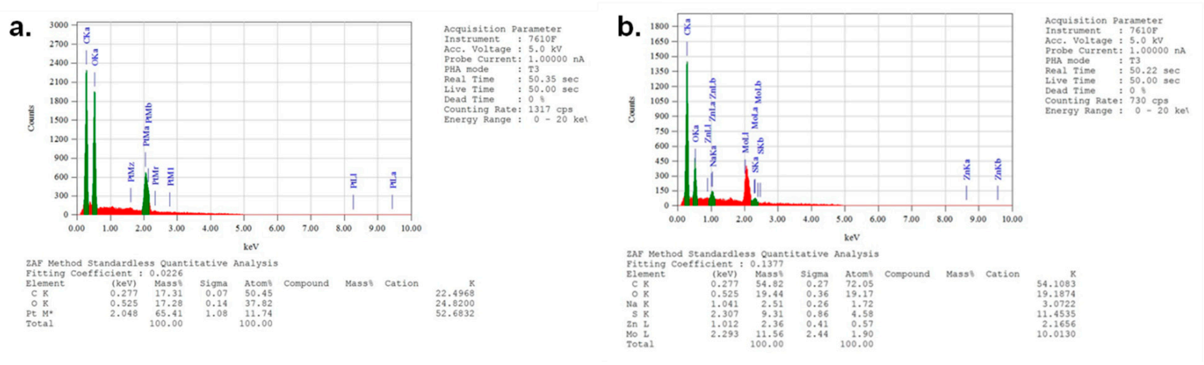

Figure S2. The EDX spectrum and composition ratio table of (a) bare unloaded PP film and (b) Dye (ICG) as a model drug loaded PP film.

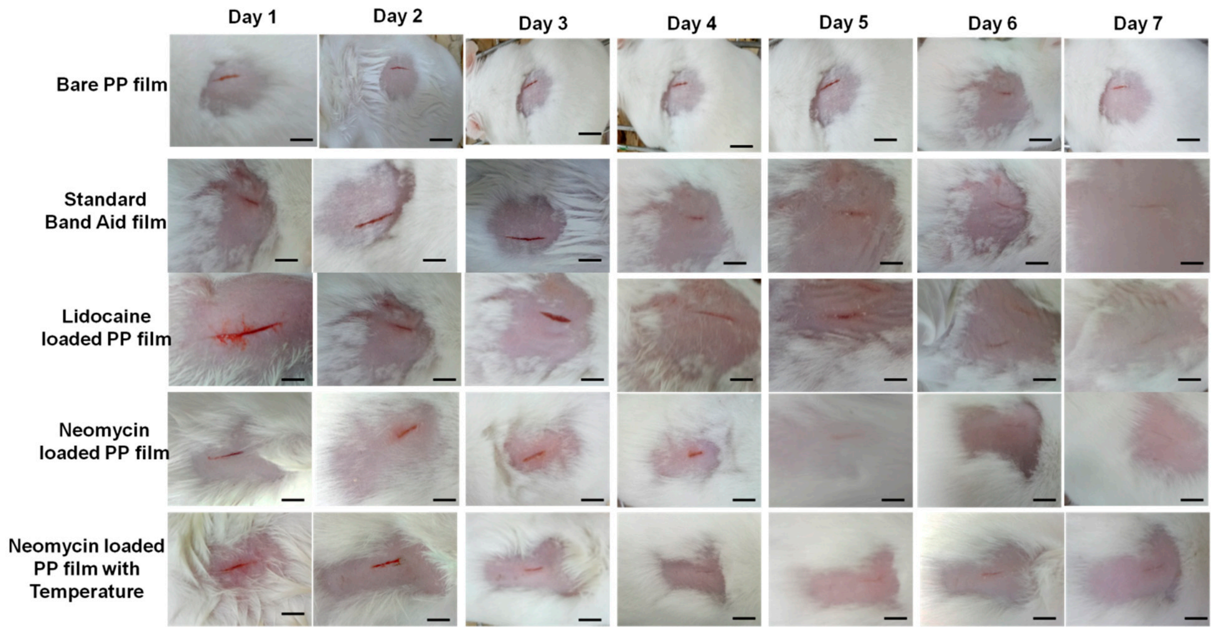

Figure S3. The images of full-thickness incision on a mice skin immediately after closure with Bare PP film, Standard Band-Aid film, Lidocaine loaded PP film, Neomycin loaded PP film, and Neomycin loaded PP film with two minutes mini-heater treatments, respectively. The digital image shows recovery after day 1 to day 7 of different treatments group (scale bar 1 cm).
